# Cases of Injuries of the Head, Treated at the Middlesex Hospital

**Published:** 1827-03

**Authors:** 


### INJURIES OF THE HEAD.

### *Cases of Injuries of the Head, treated at the* Middlesex Hospital.*

(Continued from page 117.)

It will no doubt be remarked, that those injuries of the head which have been denominated concussion and compression of the brain have many symptoms which are common to both. Indeed, as we have been considering these symptoms as arising in either instance from the same cause, the diagnosis in the early stage of such injuries must obviously be difficult. It cannot have escaped notice how ill the system, in these cases, bears the large abstraction of blood; and that it requires the utmost care, and the most mature judgment on the part of the practitioner, so to employ this remedy that it may prove safe and beneficial to his patient. The preceding cases will show that it is the inflammation of the brain with which we have to contend, and that such inflammation cannot be treated after the same manner as the idiopathic inflammation of that organ. General bloodletting cannot be so freely employed, and then it is that local depletion becomes of the utmost value. The treatment of

* In the former part of this Paper, case of R. Smith, p. 113, line 3d, for "compression" read "concussion."

apoplexy seems to stand somewhat in the same relation; for modern experience has shown that the large blood-lettings, which were formerly employed in this disease, are far from proving beneficial. The causes of this difference has been alluded to in the cases of Todd and Spirit. It would appear that, when such an important organ as the brain is much injured, the powers of life will not allow of large abstractions of blood; so that our patient may even die of inflammation of the brain without its being in our power to arrest it. Then it is that mercury, and especially calomel, may be exhibited with the greatest advantage.

There is one other circumstance which we may mention whilst speaking of the effects of blood-letting in this class of cases. We must be careful not to carry our general depletion too far; for the pulse is very apt to sink rapidly, and this sinking of the pulse is not unfrequently followed by convulsions,—a condition from which possibly, or indeed probably, our patient may not again recover. But besides this, if, in attempting to remove that degree of chronic inflammation of the brain which so often remains to be combated in the latter part of our treatment, we deplete and weaken our patient too much, we shall find that such an irritable and weakened condition of the system may be induced as to become a means of keeping up and prolonging the disease; and that, for the removal of the disease still going on in the brain, it will be necessary, at the same time that we use local depletion and counter-irritation, to support the general powers of the constitution by a mild and nutritive diet. So important do these positions appear, that we shall add another case to illustrate them.

CASE IX. *Concussion of the Brain, accompanied by Compression or some local Injury of that Organ, causing Hemiplegia.* Treated by Mr. SHAW.

John Goodison, ætatis twenty-eight, was admitted into the Middlesex Hospital, December 21st, 1826. He had fallen from a music-cart, and pitched with his head upon the pavement. He had been drinking, and when brought here was senseless: he was pale and powerless, and his pulse was small; his head rested upon his shoulder, and his eyelids dropped. The pupils were dilated, and were but little influenced by the light; the *voluntary* muscles of the eyeball were inactive, the eyeball was turned up.

When placed in bed, he immediately huddled and packed himself up into a small space, drew the bedclothes over him, and evidently wished to be left alone. He was impatient if roused, and pulled his hand forcibly away when you took it to feel the pulse. He answered questions put to him in a loud tone of voice, but his answers were short and delivered in a peevish manner. His eyelids were closed; and, with apparent perverseness, he resisted any attempts to open them, by squeezing them forcibly together.

Two small wounds of the scalp were all the external appearances of injury which could be found. When we examined the wounds, he evinced great uneasiness, and raised his hands to his head, as if to prevent further examination.

He says that he is thirsty, and assists the nurse in guiding the jug to his mouth; but, as if conscious of his own inability to hold it, he desires the nurse "not to let it go altogether:" he drinks, and afterwards resumes his former position. He lies as it were in a placid slumber, and with tranquil breathing. The head was shaved, and covered with an evaporating lotion. As we could not get him to swallow, his tongue was touched with the croton-oil, and a laxative enema was administered. Twenty leeches were applied to the head.

22d.—Is in much the same condition. He is peevish when questioned, but his answers, though short, are extremely pertinent, and evince a good deal of wit and shrewdness. If not roused, he lies in a comatose state. He had risen twice in the night, got out of bed, passed his urine, and had a plentiful evacuation from the bowels. Either time he staggered to his bed, and crept under the clothes as before. He complains of pain in the head; his pulse is weak, and rather slower than natural.

23d.—The pulse had acquired more strength. He was bled to sixteen ounces, or until the pulse fell. The nape of the neck was blistered. The bowels were kept freely open with the house-medicine. There was no alteration in the general symptoms.

24th.—When roused, he appears to be more rational than he was yesterday. Shaking his head gives him pain; the pulse is fuller.

> Sixteen ounces of blood were abstracted from the temporal artery, and the following draught was ordered to be taken three times a-day:—H. Salin. ℥ij.; Vin. Antimon. Tart. ʒj.

After the bleeding he opened his eyes; became communicative, told us his name and occupation, gave us his hand, and, when desired, put out his tongue. He could not, however, remember any thing of the accident: indeed, he knew not that any had befallen him. At times again, as if his attention was fatigued, he would relapse into his former state of somnolency.

About four P.M. (nearly two hours after the bleeding,) he had several convulsive twitches, which lasted for a few minutes. These gradually assumed a more formidable character, until the whole of the left side of the body became somewhat affected. The pulse had remained weak ever since the bleeding, but now it became very feeble and fluttering. The house-surgeon, imagining that the bleeding had been carried too far for the injured condition of the brain, and finding that the powers of the circulation were fast failing, administered a diffusible stimulant, and carefully watched its effects on the pulse. After this the patient soon recovered, and in the evening appeared to be better than hitherto.

25th.—He is quite rational: he tells us how he has passed the night, and says that he cannot sleep. He drinks continually. Bowels torpid; pulse natural. The convulsions occurred again at night.

26th.—He complains of pain in the head; pulse ninety, and weak. Bowels to be freely opened with the house-medicine. The convulsions still continue; towards midnight they became very violent.

Sinapisms to be applied to the feet.

27th.—He has lost the power of moving the left arm and leg; he cannot retract the left cheek, nor can he forcibly close the eye-lid. When asked to move his left hand, he lifts it with the right. The convulsive motions of the voluntary muscles are confined entirely to the paralysed side; but it is curious to observe that the spasmodic action of the respiratory muscles is equal on both sides. This shows well the combination of the respiratory muscles, and the indissoluble connexion which exists between the function of respiration on either side. He is almost constantly convulsed; the intervals are short, but perfect. The sweat stands upon his brow in large drops, and his body is drenched with perspiration. He is quite insensible during the fit. In the evening the pulse became full, and the carotids beat strongly. Eight ounces of blood were taken from the temporal artery: the pulse then began to faulter, and the bleeding was stopped.

A blister to be applied to the nape of the neck.

28th.—He is evidently worse: he faulters in his speech, and he passes his feces and urine in bed. The fits continue. The sinapisms had produced but little effect. The blistering iron was applied to the inside of the legs: this produced some irregular movements in the paralysed limb.

31st.—Has continued much the same since our last report. Yesterday he was convulsed for three hours, without any perceptible remission, and immediate fears for his life were entertained. To-day there is more excitement, and evidently more action in the vascular system of the brain.

Twelve leeches were applied to the head; and two grains of Calomel were ordered to be taken three times a-day.

January 2d.—He is much improved: he has partially recovered the use of his left arm and leg; he can now close the left eyelid firmly, and can draw the angles of the mouth to either side. His fits have been less frequent; he is far more rational; but he still passes his feces and urine in bed. Gums not affected.

10th.—The improvement in this man's condition since the first exhibition of the mercury, but especially since the system has been brought under its influence, is truly remarkable. The paralysis has been in a great measure removed, and the functions of the sensorium have been partially restored. He remains in a very irritable and weak condition, and continues to complain of pain in his head; and his other symptoms are indicative of some inflammation still existing in the brain. He was allowed a more nutritive diet.

This alteration in diet, together with a perseverance in counter-irritation, and a moderate use of mercury, proved highly beneficial, and led to a perfect cure.

[To be continued.]

